# Laser Light-field Fusion for Wide-field Lensfree On-chip Phase Contrast Microscopy of Nanoparticles

**DOI:** 10.1038/srep38981

**Published:** 2016-12-13

**Authors:** Farnoud Kazemzadeh, Alexander Wong

**Affiliations:** 1 Department of Systems Design Engineering, University of Waterloo, Waterloo, Ontario, N2L 3G1, Canada

## Abstract

Wide-field lensfree on-chip microscopy, which leverages holography principles to capture interferometric light-field encodings without lenses, is an emerging imaging modality with widespread interest given the large field-of-view compared to lens-based techniques. In this study, we introduce the idea of laser light-field fusion for lensfree on-chip phase contrast microscopy for detecting nanoparticles, where interferometric laser light-field encodings acquired using a lensfree, on-chip setup with laser pulsations at different wavelengths are fused to produce marker-free phase contrast images of particles at the nanometer scale. As a proof of concept, we demonstrate, for the first time, a wide-field lensfree on-chip instrument successfully detecting 300 nm particles across a large field-of-view of ~30 mm^2^ without any specialized or intricate sample preparation, or the use of synthetic aperture- or shift-based techniques.

Phase contrast microscopy was introduced as a means of observing objects with light transparent properties^1^. Bright-field microscopy of such objects tend to produce low contrast images since the probing light and the sample being imaged do not undergo a very strong interaction. Phase contrast microscopy, however, allows for visualization of the optical path length differences that the probing light would experience as the result of the interaction with the sample, thereby producing high contrast images of such samples. Despite the aforementioned benefit of phase contrast microscopy, it nevertheless suffers from the same shortcomings of bright-field microscopy such as limited field-of-view (FOV), limited resolution capability, and high complexity of the design and operation of the instrument.

Wide-field lensfree on-chip microscopy, where holography principles are used to capture interferometric light-field encodings without lenses, has become an interesting pathway toward addressing the shortcomings of lens-based techniques^2^,^3^,^4^,^5^,^6^,^7^,^8^,^9^,^10^,^11^,^12^,^13^,^14^,^15^,^16^,^17^,^18^,^19^,^20^,^21^,^22^. At the core, these lensfree on-chip instruments are conceptually simple and offer extremely large FOV in comparison to lens-based instruments. Given that the sample slide sits on top of the detector in a lensfree on-chip setup, the pixel pitch of the detector can affect the ability to detect particles at the nanometer scale. Synthetic aperture- or lateral shift-based techniques^7^,^8^,^9^,^11^,^23^,^24^ have been proposed for improving resolution, though such techniques tend to increase the hardware complexity and decrease performance tolerance of the instrument. More recently, multi-wavelength illumination-based techniques^17^,^20^,^21^,^22^,^25^ have also showed considerable promise for improving resolution.

A particular area of interest for employing wide-field lensfree on-chip microscopy is for detecting particles at the nanometer scale. Super-resolution microscopy^26^,^27^, atomic force microscopy^28^, and electron microscopy^29^ have been the mainstays for detecting particles at the nanometer scale but are limited by very high instrumentation cost and complexity, narrow FOV, and low imaging throughput due to the complexity of the imaging process. The ability to harness the principles behind wide-field lensfree on-chip microscopy for detecting nanoparticles holds considerable promise for greatly reducing imaging cost and complexity while improving image throughput and FOV, thus enabling comprehensive, large scale studies to be more readily achieved. Recent results are quite promising^30^,^31^, demonstrating the ability to detect nanoparticles of various sizes through the use of some additional sample preparation (e.g. ref. 30 requires the use of biocompatible wetting films to self-assemble aspheric liquid nanolenses around individual nanoparticles) which may increases imaging complexity. As such, a method to achieve wide-field lensfree on-chip microscopy for detecting nanoparticles without the need for additional specialized sample preparation, or the use of synthetic aperture- or lateral shift-based techniques is highly desired.

In this study, we introduce the idea of laser light-field fusion phase contrast microscopy for detecting nanoparticles. Inspired by our earlier, preliminary exploration into spectral light-field fusion^20^,^21^,^22^, laser light-field fusion phase contrast microscopy involves the acquisition and fusion of interferometric laser light-field encodings using a lensfree, on-chip setup with laser pulsations at different wavelengths to produce marker-free phase contrast images, as shown in Fig. 1. The proposed instrument allows us to detect nanoparticles using a lensfree, on-chip setup, thus reducing imaging cost and complexity compared to other solutions. As proof of concept, we demonstrate, for the first time, a lensfree on-chip instrument capable of detecting 300 nm particles across a large field-of-view of ~30 mm^2^ without utilization of specialized or intricate sample preparation, or the use of synthetic aperture- or lateral shift-based techniques.

## Materials and Methods

### Imaging apparatus

**Figure 1:**
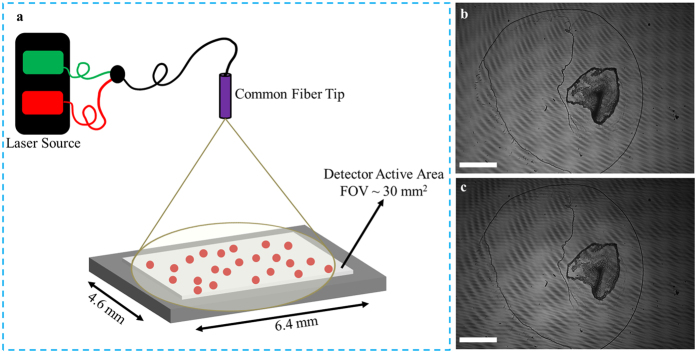
The proposed instrument along with the imaging results. (**a**) The schematic of the instrument showing the single-mode fiber optic cable which is used by two-channel pulsed laser light source; the size of the detector array which is the total field-of-view of the instrument is noted. (**b** and **c**) The captured interferometric light-field encoding (*λ*_1_ = 531.9 nm (**b**) and *λ*_2_ = 638.3 nm (**c**)), with the scale bars denoting 1 mm.

The proposed wide-field lensfree on-chip phase contrast microscopy instrument used for this study (shown in Fig. 1) can be described as follows. A two-channel pulsed laser light source is used, with central wavelengths at *λ*_1_ = 531.9 nm and *λ*_3_ = 638.3 nm, and the spectral bandwidth being 1 nm. The pulsed laser light source was programmed to pulse with an alternating wavelength pulse sequence, where the duration of the pulsations are configured such that the signal observed on the detector is maximized while mitigating pixel saturation. The pulsed laser light source is coupled to a single-mode fiber optic cable to illuminate the sample. The detector readout is synchronized to the pulse sequence for rapid and seamless interferometric light-field encoding acquisitions at the two wavelengths of the laser light source. The exposure time was <1 ms for each wavelength, resulting in interferometric light-field encoding acquisitions using the proposed instrument taking less than ~2 ms (equivalent to >500 frames per second).

The sample being imaged is placed on a #1 microscope cover slip with a thickness of ~145 *μ*m, which sits directly on the detector. Interferometric light-field encoding acquisitions of the sample being imaged at different wavelengths are made by the detector using a 3840 × 2748 pixel CMOS sensor array with a pixel pitch of 1.67 *μ*m. The FOV of the device is determined based the active sensor size and is ~30 mm^2^. The captured interferometric light-field encoding, denoted by *g*_*x*,*y*,*λ*_ with *λ* denoting wavelength, is then sent to the digital signal processing unit, where numerical laser light-field fusion is performed to reconstruct a fused phase contrast image *r*_*x*,*y*,*z*_.

In this study, the measurement instrument was characterized based on a number of acquisitions of point source targets to determine the aberration transfer function at each wavelength (denoted by 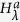) of the pulsed laser light source to account for differences at different wavelengths.

For comparison purposes, a reference lensfree on-chip instrument capturing interferometric light-field encodings at *λ* = 531.9 nm using the aforementioned imaging apparatus (with a single-channel laser light source is used instead) was also evaluated in this study.

### Image Reconstruction

The numerical laser light-field fusion performed on the digital signal processing unit to reconstruct fused phase contrast images from measurements made by the proposed instrument can be described as follows. The captured interferometric laser light-field encoding *g*_*x*,*y*,*λ*_ encapsulates unique diffraction behaviour at different wavelengths *λ*, which we will leverage to achieved improved image quality beyond that can be achieved using a single wavelength. Let us formulate a fused laser object light-field *q*_*x*,*y*,*z*_ as a subspace projection of the laser object light-field *f*_*x*,*y*,*z*,*λ*_,


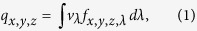


where *v*_*λ*_ denotes the *λ*-associated coefficient of the largest eigenvector of the correlation matrix of *f*_*x*,*y*,*z*,*λ*_, thus taking into account the correlation structure across *λ*. Given that the instrument described captures *g*_*x*,*y*,*λ*_, not *f*_*x*,*y*,*z*,*λ*_, one must devise a mechanism to numerically estimate *q*_*x*,*y*,*z*_ given *g*_*x*,*y*,*λ*_.

Let us now model the laser object light-field *f*_*x*,*y*,*z*,*λ*_ and the interferometric laser light-field encoding *g*_*x*,*y*,*λ*_ as probability distributions. To estimate *q*_*x*,*y*,*z*_, we now wish to compute a subspace projection of the most probable laser object light-field *f*_*x*,*y*,*z*,*λ*_ given *g*_*x*,*y*,*λ*_, with *a priori* knowledge related to *f*_*x*,*y*,*z*,*λ*_ and the aberration transfer function (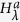), as well as the Rayleigh-Sommerfeld diffraction transfer function (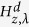),


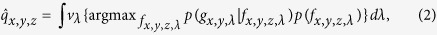


where *p*(*g*_*x*,*y*,*λ*_|*f*_*x*,*y*,*z*,*λ*_) denotes the likelihood of *g*_*x*,*y*,*λ*_ given *f*_*x*,*y*,*z*,*λ*_ and *p*(*f*_*x*,*y*,*z*,*λ*_) denotes the prior of *f*_*x*,*y*,*z*,*λ*_. According to quantum photon emission statistics, one can express *p*(*g*_*x*,*y*,*λ*_|*f*_*x*,*y*,*z*,*λ*_) as


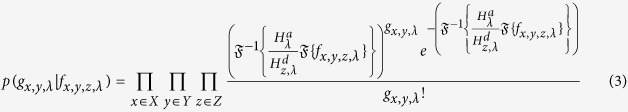


where 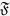 and 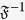 denotes the forward and inverse Fourier transform, respectively. Modeling *f*_*x*,*y*,*z*,*λ*_ as a nonstationary process, one can express *p*(*f*_*x*,*y*,*z*,*λ*_) as


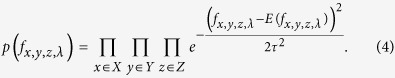


where *E*(*f*_*x*,*y*,*z*,*λ*_) denotes the nonstationary expectation and *τ*^2^ denotes the variance. To reconstruct the fused phase contrast image *r*_*x*,*y*,*z*_, the computed 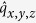 is phase-shifted by 90° at the zeroth frequency, and the amplitude of this phase-shifted 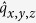 is then taken as *r*_*x*,*y*,*z*_^1^.

An expectation maximization for MAP estimation is used to solve Eq. 2, and is performed until convergence.

## Results

**Figure 2:**
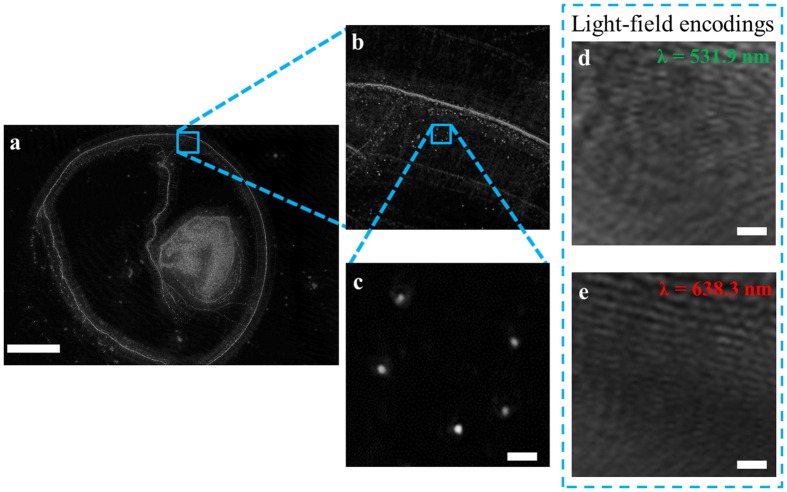
The reconstructed phase contrast microscopy image of 500 nm nanospheres. (**a**) The full FOV image of the detector, scale bar denotes 1 mm. (**b**) A zoomed-in region of **a**. (**c**) Further zoomed-in of a specific region in **b** showing a ‘U’ arrangement of 500 nm nanospheres. (**d** and **e**) The captured light-field encodings at *λ*_1_ = 531.9 nm (**d**) and *λ*_2_ = 638.3 nm (**e**) used to obtain **c**. The scale bars in **c, d, e** all denote 2 *μ*m.

The capability of the proposed instrument in detecting particles at the nanometer scale is first demonstrated in Fig. 2, where a phase contrast image of a sample consisting of polystyrene nanospheres (Fluoresbrite, Polysciences, Inc., USA) is shown in Fig. 2a. Upon closer examination, through two zoom levels, Fig. 2b and c, a phase contrast image of an isolated cluster of five 500 nm nanospheres are discovered, arranged in a ‘U’ formation. The light-field encoding captured at *λ*_1_ = 531.9 nm and *λ*_2_ = 638.3 nm are shown in Fig. 2d and e, respectively.

**Figure 3:**
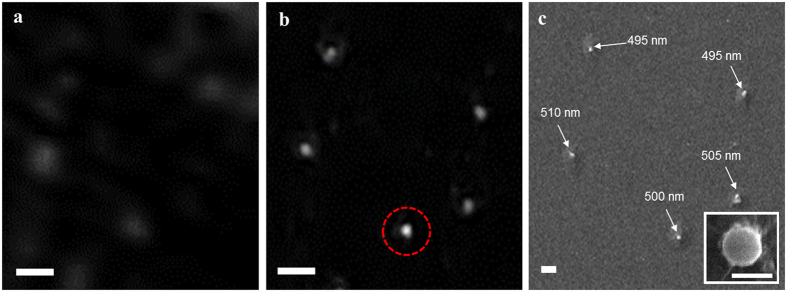
Imaging capability of the proposed system using a collection of 500 nm nanospheres with SEM validation. (**a**) The phase contrast image containing five nanospheres arranged in a ‘U’ formation obtained using the reference lensfree on-chip instrument capturing interferometric light-field encodings at *λ* = 531.9 nm. (**b**) The phase contrast image obtained using the proposed instrument containing five nanospheres arranged in a ‘U’ formation. (**c**) The SEM image of the corresponding region with the size of the nanospheres noted on the image (with the inset showing a magnification of the nanospheres circled in red). The scale bars in **a**, **b**, and **c** denote 2 *μ*m, with the scale bar in the inset of **c** denoting 500 nm.

Scanning electron microscopy (SEM) (MERLIN, Carl Zeiss AG, Germany) was used to verify the imaging results captured by our instrument, as shown in Fig. 3. The corresponding location of the ‘U’ formation of the nanospheres are shown using a reference lensfree on-chip instrument capturing interferometric light-field encodings at *λ* = 531.9 nm (see Fig. 3a), the proposed laser light-field fusion phase contrast microscopy instrument (see Fig. 3b), and SEM (see Fig. 3c). The sizes of the nanospheres, determined based on the largest distance from one side of the particle to the other side of the particle in the SEM image, are consistent within the manufacturer’s fabrication tolerance of 500 ± 3% nm^32^. A number of observations can be made from Fig. 3 with regards to the reference lensfree on-chip instrument and the proposed laser light-field fusion phase contrast microscopy instrument. It can be observed that the same nanospheres observed by the SEM cannot be detected using the reference lensfree on-chip instrument capturing interferometric light-field encodings at *λ* = 531.9 nm. On the other hand, it can be observed that the same nanospheres observed by the SEM have been detected with the proposed laser light-field fusion phase contrast microscopy instrument, thus demonstrating its ability to detect particles that are 495 nm in size as well as achieve superior performance to the reference lensfree on-chip instrument. Furthermore, it can be observed that differentiating the size variability of these nanospheres is beyond the capability of the proposed instrument as such variability is beyond the capabilities of the instrument.

It can also be observed that the image quality achieved using the proposed laser light-field fusion phase contrast microscopy instrument is high at a detection signal-to-noise ratio (SNR) of 33.35 dB, achieved by only capturing a single interferometric light-field encoding at a given wavelength in the current setup.

**Figure 4:**
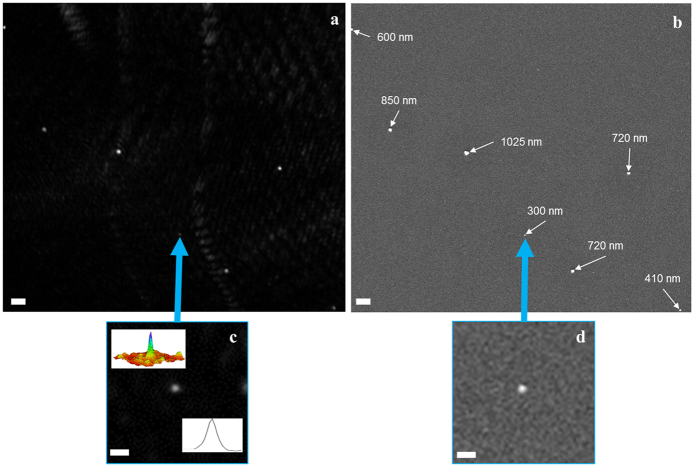
Demonstration of the imaging capability using a collection of particles of different sizes with SEM validation. (**a**) A select location of a ~30 mm^2^ FOV acquired by the proposed instrument containing seven particles of varying sizes. (**b**) The SEM image of the same FOV. (**c**) The zoomed-in image of the 300 nm particle with the cross-sectional profile and the surface intensity map inset. (**d**) The SEM verification image of the 300 nm particle. The scale bar in **a** and **b** denote 3 *μ*m and in **c** and **d** denote 1 *μ*m.

The capability of the proposed instrument was further demonstrated using a sample consisting of a mixture of particles of different sizes that was prepared for imaging. A zoomed-in region of this sample is shown in Fig. 4a and b imaged by the proposed instrument and an SEM, respectively. There are six different sizes of particles in this region varying from 300 nm to ~1 *μ*m, again determined based on the largest distance from one side of the particle to the other side of the particle in the SEM image. The size of these particles are verified using the SEM and the corresponding particles are observed using the proposed instrument. A number of observations can be made from Fig. 4. It can be observed that the smallest particle, which is 300 nm in size, is detected using both the SEM and the proposed laser light-field fusion phase contrast microscopy instrument, with Fig. 4c and d showing this particle in isolation for the proposed instrument and SEM, respectively. It can also be observed from the cross-sectional profile and the intensity surface of the particle (see inset of Fig 4c) that a positive detection of the 300 nm particle is demonstrated, with a detection SNR of 29.61 dB. Furthermore, it can be observed that the size variation of different sized particles are explicitly reflected in the phase contrast image, as their imaged size tends to reflect the actual size. These results demonstrate as a proof-of-concept the capabilities of the proposed instrument, which is beyond existing lensfree on-chip instruments that do not require any specialized or intricate sample preparation, or the use of synthetic aperture- or shift-based techniques.

## Discussion

We introduced an wide-field lensfree on-chip phase contrast microscopy instrument for detecting particles at the nanometer scale. As proof-of-concept, the capability of the proposed instrument is demonstrated by imaging particles which are 300 nm in size. These experimental results demonstrate that the unique diffraction behavior captured in the acquired interferometric laser light-field encodings at different wavelengths can be leveraged through a fusion process to achieve improved image quality in a lensfree on-chip instrument beyond that can be achieved using a single wavelength.

It is important to note that the proposed wide-field lensfree on-chip instrument does not require specialized sample preparation, or the use of synthetic aperture- or lateral shift-based techniques to accomplish detection of nanoparticles. What this implies is that the proposed instrument has low instrumentation complexity and cost, and is easy to operate and maintain, thus allowing for democratization and proliferation of such instruments in healthcare, industry, education, and research. The interferometric light-field encoding acquisitions made by the proposed instrument take less than ~2 ms (equivalent to >500 frames per second), thus enabling observations of highly time-resolved dynamic systems or transient phenomena at the nanometer scale, such as for the study of motion dynamics of colloidal nanoparticles.

A limitation with the setup of the proposed instrument used in this current study is that the number of wavelengths used is restricted to two different laser wavelengths, which can be addressed in future studies through the incorporation of a tunable laser to potentially enable further improvements in image quality.

## Conclusions

We introduced an wide-field lensfree on-chip phase contrast microscopy instrument capable of detecting particles at the nanometer resolution. The instrument does not require hologram magnification, specialized sample preparation, or the use of synthetic aperture- or lateral shift-based techniques to accomplish detection of nanoparticles. Interferometric light-field encoding acquisitions using the proposed instrument take less than ~3 ms (equivalent to >300 frames per second), which would allow for observation of highly time-resolved dynamic systems or transient phenomena, at the nanometer scale with superior image quality, SNR > 29 dB. Additionally, the proposed instrument is extremely simple and economical to implement allowing for democratization and proliferation of such systems at every level of healthcare, industry, education, or research.

## Additional Information

**How to cite this article**: Kazemzadeh, F. and Wong, A. Laser Light-field Fusion for Wide-field Lensfree On-chip Phase Contrast Microscopy of Nanoparticles. *Sci. Rep.* **6**, 38981; doi: 10.1038/srep38981 (2016).

**Publisher’s note:** Springer Nature remains neutral with regard to jurisdictional claims in published maps and institutional affiliations.
